# COVID-19 vaccine acceptance and hesitancy in Ghana: A systematic review

**DOI:** 10.1371/journal.pone.0305993

**Published:** 2024-06-25

**Authors:** Godwin Banafo Akrong, Rosemond Akpene Hiadzi, Antonia Bernadette Donkor, Daniel Kwasi Anafo

**Affiliations:** 1 Management Science and Economics, University of Electronic Science and Technology of China, Chengdu, China; 2 School of Information Studies, McGill University, Montreal, Canada; 3 Sociology Department, University of Ghana, Accra, Ghana; 4 Balme Library, University of Ghana, Accra, Ghana; 5 Office of Research, Innovation and Development (ORID), University of Ghana, Accra, Ghana; Nurses’ and Midwives Training College, GHANA

## Abstract

The propensity to accept vaccines and factors that affect vaccine acceptance and hesitancy will determine the overall success of the COVID-19 vaccination program. Therefore, countries need to understand the factors that influence vaccine acceptance and hesitancy to prevent further future shocks, and it is necessary to have a thorough understanding of these factors. As a result, this study aims to review selected published works in the study’s domain and conduct valuable analysis to determine the most influential factors in COVID-19 vaccine acceptance and hesitancy in Ghana. The review also explored the acceptance rate of COVID-19 vaccines in Ghana. We selected published works from 2021 to April 2023 and extracted, analyzed, and summarized the findings based on the key factors that influence COVID-19 vaccine acceptance and hesitancy in Ghana, the acceptance rate in Ghana, the demographic factors that are often examined, and the study approach used to examine these factors. The study found that positive vaccination perception, safety, belief in vaccine efficacy, knowledge of COVID-19, and a good vaccine attitude influence COVID-19 vaccine acceptance in Ghana. The negative side effects of the vaccines, mistrust in the vaccine, lack of confidence in the vaccine’s safety, fear, and spiritual and religious beliefs all played significant roles in influencing COVID-19 vaccine hesitancy. For this study, the COVID-19 acceptance rates observed in the reviewed articles ranged from 17.5% to 82.6%. The demographic parameters frequently included in these studies that have a significant impact include educational attainment, gender, religious affiliation, age, and marital status. The positive perceptions of the COVID-19 vaccine and concerns about its negative effects influenced Ghanaians’ acceptance and hesitancy.

## 1. Introduction

The COVID-19 pandemic has emerged as a worldwide public health crisis, presenting substantial challenges to healthcare systems and governments [1]. To mitigate the spread of the pandemic, public health and social distancing measures were implemented, such as improved hand hygiene practices, adherence to physical distancing guidelines, and the use of personal protective equipment.

Following the COVID-19 pandemic, the Africa Center for Disease Control implemented the SARS-COV-2 Development and Access Strategy in 2020. The primary objective of this strategy was to achieve vaccination coverage of at least 60% among the African population by 2022 to attain herd immunity [2, 3]. According to the World Health Organization (WHO) [4], a total of 53 out of 54 African countries have initiated the COVID-19 immunization campaign. As of 01/ 04/ 2024, data from the WHO shows that these countries have collectively received 1,114,404,185 cumulative doses. Out of this total, the WHO has administered 865,103,036 doses or approximately 78%. The WHO reports that the vaccination rate in Africa stands at 62 doses per 100 individuals. Approximately 39.9% of the African population, or 559,869,883 individuals, have received at least one dose of the vaccine, according to the most recent data (01/04/2024). Furthermore, 33.1% of the population, equivalent to 465,429,347 individuals, has completed the primary series of vaccinations. This implies the need to improve vaccination coverage among Africans, especially for those who have only received a single dose and those who have not received any vaccinations. Encouraging these individuals to complete their vaccination regimen would go a long way toward helping the African Center for Disease Control achieve its 2020 target. While the World Health Organization declared the COVID-19 epidemic over on May 5, 2023, they stressed that it remained a worldwide health danger [5]. Thus, the 2020 goal of the African Center for Disease Control remains relevant.

This observation underscores the importance for African nations to prioritize the study of factors influencing individuals’ acceptance or hesitancy towards COVID-19 vaccines. It is crucial to understand these factors, as they are believed to impact both individuals who have received only one dose and those who have not yet received the vaccine. Ghana, located in West Africa, is actively participating in this endeavor. Based on the WHO’s report from 01/04/2024, it is evident that all 17 West African countries have initiated the COVID-19 immunization campaign [4]. In total, these countries have received approximately 337,393,634 vaccine doses, with 266,775,705 doses (79%) administered. Notably, the vaccination rate in West Africa is 57 doses per 100 people. Notably, 38.2% of the population from West African countries has received at least one dose of the COVID-19 vaccine. Furthermore, around 32.1% of the population, which amounts to 150,175,825 individuals, has completed the initial series of vaccinations.

Nevertheless, it is imperative to implement additional steps to ensure that those who have received only one dose of the vaccine follow through with completing the whole vaccination regimen. Moreover, it is crucial to establish mechanisms that encourage individuals who have not yet received any doses of the vaccine to do so. The primary objective of this study is to carry out an in-depth review, analysis, and assessment of the acceptability and hesitancy of the COVID-19 vaccine in Ghana. This is intended to help determine the factors that influence COVID-19 vaccine acceptance and hesitancy, with a focus on Ghana.

According to the WHO [4], as of April 1, 2024, Ghana had received a total of 34,047,598 cumulative doses of vaccines. Out of these doses, they have successfully administered 25,624,828, which accounts for almost 75% of the total received. Therefore, approximately 8,422,770 doses remain unadministered. However, additional data from the WHO, updated on April 1, 2024, shows that 13,864,186 people, or roughly 43.7% of Ghana’s population, have received at least one dose of the vaccine. This indicates that a greater number of vaccines will be required for individuals to achieve full COVID-19 vaccination, despite 10,780,003 individuals having already finished the vaccination process. Ghana stands out for its high vaccine administration rate, administering 81 doses per 100 individuals in the population. However, the objective is to achieve widespread acceptance of the COVID-19 vaccination throughout the general population while also identifying the variables that hinder its acceptance. Therefore, the current systematic review is necessary to identify the factors that researchers have identified as significant contributors to COVID-19 vaccine acceptance in Ghana, as well as the factors that contribute to vaccine hesitancy among the Ghanaian population. This can serve as a foundation for motivating people who have not yet received the vaccine or their second dose.

Ghana mostly used static, outreach, mobile, campout, or a combination of these methods to distribute COVID-19 vaccinations [6]. The vaccination sites were health centers, hospitals, outreach points, pharmacies, markets, and other public areas serving specific target populations. Ghana’s COVID-19 immunization program required stakeholder engagement [7]. WHO also brought together Ghanaian medical, pharmaceutical, nursing, midwifery, allied health, and psychological societies to start a larger conversation. The Ghana Academy of Arts & Sciences, Parliamentary Leadership, Plenary, and religious groups all participated. Ghana meticulously prepared and vigorously implemented communication and demand-development strategies to emphasize vaccine safety and efficacy. Ghana also adopted proactive measures to combat disinformation and vaccine reluctance. Ghana successfully divided the population by risk level, disease severity, ability to continue business as usual, and national security [6]. These segments determined the vaccination allocation. Resilient data and coverage monitoring systems ensure process efficiency.

District Health Information Software 2 (DHIS2), specifically an e-tracker for individual records (which includes sending reminders to customers for further dosages) and the platform for aggregated data, helped to accomplish this [8]. Each vaccination prompted the issuance of a vaccination card. According to the implemented procedures, there was an adequate supply of vaccines in Ghana during the study period. According to Africa Centres for Disease Control and Prevention (CDC) data [9], Ghana had an adequate supply of AstraZeneca, Sputnik V, Pfizer BionTech, J&J, and Moderna vaccines, as illustrated in the S1 Appendix. According to WHO data, 80% of healthcare workers, 41.70% of older adults (1,469,207), 32% of people with comorbidities (693,121), and 425,174 under-17 years in Ghana had completed the primary COVID-19 vaccine series as of April 1, 2024 [4].

During the COVID-19 vaccine era, Amponsah-Tabi et al. [10] investigated how media knowledge influenced vaccination acceptance. They emphasized that individuals with prior vaccination knowledge or education were more likely to accept the COVID-19 vaccine due to media awareness. Amponsah-Tabi et al. also observed that rural Ghanaians’ acceptance, understanding, and perception of the COVID-19 vaccination are inadequate and recommended educational outreach to rural residents about the vaccine’s benefits. Their study found that people who had previously received vaccinations were more likely to accept the COVID-19 vaccine, and they recommended using national vaccination programs like the Hepatitis B program to increase COVID-19 vaccine acceptance. While studying Ghanaian teenagers’ vaccine uptake, Adjaottor et al. [11] recommended that parents and guardians should play a larger role in helping their children choose the correct COVID-19 vaccine. Furthermore, the majority of research on Ghana’s acceptance of the COVID-19 vaccine took place before the vaccination campaign [12]. The availability of vaccines may change people’s decisions. These studies indicated that the Ghanaian government should promote positive attitudes about vaccinations, increase the sense of virus danger, and educate the population about vaccination’s benefits.

Healthcare practitioners are at high risk for COVID-19 infection and death, according to Alhassan et al. [13]. Thus, they must accept and use the COVID-19 immunization to protect their health and safety. Their study also concluded that younger healthcare workers, non-Christians, and those in religious facilities were more likely to receive the COVID-19 vaccine. According to Forkuo et al. [14], understanding COVID-19 and the vaccines associated with it is crucial to vaccination acceptance in Ghana. In addition, COVID-19 vaccine coverage campaigns should emphasize safety, side effects, and management. By addressing the misunderstandings associated with these factors, one can actively contribute to the elimination of erroneous beliefs that permeate society at large.

Further research suggests that the health management team can reduce vaccine hesitancy by focusing on minimizing the negative impact of the media and other factors, such as fear and mistrust in leadership [15]. Additionally, research has shown that older age groups in Ghana exhibit a reduced inclination to hesitate in getting vaccinated, while males tend to display more uncertainty about obtaining the vaccination compared to females [16]. Even so, the study found no significant association between the propensity to vaccinate and variables such as place of residence or educational attainment. Alhassan et al. [17] acknowledged that fear, uncertainty, conspiracy theories, and safety concerns provide substantial obstacles to the effective adoption of the vaccine if not adequately addressed. Thus, as per the findings of Botwe et al. [18], safety education should aim to alleviate the concerns of individuals who have experienced adverse reactions to previous vaccines, those who hold the belief that vaccination leads to infertility, and those who have underlying medical conditions but are uncertain about their eligibility for vaccination.

Amo-Adjei et al. [19] reported that individuals’ belief in the vaccine’s efficacy and safety had a stronger predictive power for acceptance than their socioeconomic factors. Moreover, Amo-Adjei et al. contend that skepticism towards both domestic and international political leaders, a conviction in supernatural safeguarding, and a perceived ignorance of the intricacies of vaccine manufacturing all contribute to the refusal of vaccines. In addition, Mohammed et al. [20] concluded that many characteristics serve as significant predictors of individuals" probability of receiving the COVID-19 vaccine. The factors identified by Mohammed et al. included people aged 25 to 45, people over 45, males, and Christians. Those who chose not to receive the vaccine cited concerns regarding the perceived rapid development and approval process, potential immediate side effects, and unforeseen long-term consequences.

Despite the abundance of surveys and literature on the factors influencing the acceptance and hesitancy of COVID-19 vaccines, there is a lack of comprehensive systematic reviews in the Ghanaian context of vaccine acceptance that aim to identify the factors influencing both the acceptance and hesitancy of COVID-19 vaccines. According to the evaluation checklist by Kitchenham et al. [21], it was clear that a significant number of researchers conducting studies in Ghana mostly provided a narrative review instead of a systematic review. Their reviews encompassed broad insights into the differing determinants of COVID-19 vaccine acceptance and the factors contributing to COVID-19 hesitancy. Moreover, it is crucial to emphasize that there is presently no existing systematic review that examines various factors influencing the acceptance or hesitancy of the COVID-19 vaccine in a single paper.

This knowledge gap serves as motivation for our study, as previous studies have aimed to offer a comprehensive review, analysis, and evaluation of the patterns of COVID-19 vaccination uptake and hesitancy, specifically within Ghana. Researchers have conducted multiple systematic studies to comprehensively examine global data on COVID-19 epidemics and the determinants of national COVID-19 vaccination uptake [22–25]. The existing knowledge in the field influenced the decision to conduct a systematic review.

This paper organizes its subsequent sections as follows: The method section provides a comprehensive account of the methodology employed in the present review. The results section also presents the findings and analysis. Subsequently, the discussion section delves into the implications and potential areas for future research. Finally, we draw conclusions based on the findings.

## 2. Methodology

The current study provides a contemporary overview and evaluation of several key aspects, including (1) the factors contributing to COVID-19 vaccine acceptance in Ghana; (2) the factors contributing to COVID-19 vaccine hesitancy; (3) the most frequently assessed demographic profile, and its association with COVID-19 vaccine acceptance and hesitancy in Ghana; (4) the predominant study methodology employed in this field; and (5) the acceptance rate of COVID-19 in Ghana. To determine the eligibility of the research questions for the current systematic review study, the researchers (GBA and RAH) utilized the PEO (Population, Exposure, and Outcomes) framework commonly used in systematic reviews [26]. Table 1 presents the framework, while Table 2 showcases the five research questions formulated for this systematic literature review. The Preferred Reporting Items for Systematic Review and Meta-Analysis (PRISMA) guidelines guided the conduct of the systematic review. The review encompassed the identification, screening, and inclusion stages as per the recommended protocols [27].

**Table 1:** PEO framework for determining the eligibility of the research questions.

Criteria	#	Determinant
Population	RQ3	The group encompasses Ghanaians, health care workers, Ghanaian citizens over the age of 18, adult Ghanaians, registered radiographers, OPD attendants, government officials, community leaders, residents in both rural and urban areas, parents and guardians, and individuals of both genders. There are no limitations to ethnic group, religion, educational background, marital status, and experience.
Exposure	RQ1, RQ2,	Positive for COVID-19, SARS-CoV-2 infections, presence of symptoms associated with SARS-CoV-2 infection, acceptance of vaccine, vaccine hesitancy
Outcomes	RQ1, RQ2, RQ4, and RQ5	Acceptance and Hesitancy: These include variables that have the potential to either act as a barrier or a facilitator of COVID-19 vaccine hesitancy and acceptance. Anticipated determinants include the safety of the vaccine, trust in its effectiveness, knowledge of COVID-19, a positive attitude towards the vaccine, protective measures for oneself and family, gender, and religious affiliation. People harbor mistrust towards the vaccine, lack confidence in its safety, experience fear and uncertainty, entertain conspiracy theories, and express concerns about its impact on the Ghanaian race.
		Study approach: qualitative, quantitative, or mixed.
		Vaccine acceptance rate

**Table 2:** Systematic review questions.

#	Question	Objective
RQ1	What are the factors that contribute to the acceptance of the COVID-19 vaccine in Ghana?	Identify the factors that contribute to the COVID-19 vaccine acceptance in Ghana.
RQ2	What are the contributing causes of COVID-19 vaccine hesitancy in Ghana?	Explore the factors that contribute to Ghana’s COVID-19 vaccination hesitancy.
RQ3	Which demographic factors are commonly evaluated while determining the factors that influence the acceptance and hesitancy of the COVID-19 vaccine in Ghana?	Review the demographic factors that are typically considered when determining the factors that influence the acceptance and hesitancy of the COVID-19 vaccine in Ghana.
RQ4	What are the main study approaches utilized in examining the factors influencing the acceptance and hesitancy of the COVID-19 vaccine in Ghana?	Determine the most prevalent study methodology utilized by researchers in determining the factors that affect the acceptance and hesitancy of COVID-19 in Ghana.
RQ5	What is the COVID-19 vaccine acceptance rate in Ghana?	Examine the rate of COVID-19 vaccine acceptance in Ghana.

Initially, we developed specific research questions that aligned with the objective of this study. The next stage entails formulating a search strategy, which includes selecting search terms and suitable search engines to retrieve relevant literature for further investigation. The researchers (GBA, RAH, ABD, and DKA) established a study selection criterion to identify the relevant studies that address the research questions. To enhance the study selection criteria, we conducted a preliminary pilot study selection process. Afterward, we implemented some quality checklists to assess relevant studies as part of the quality evaluation procedure. The collected data was synthesized. The nature of the data and the research questions addressed during the data collection process guided the selection of suitable synthesis approaches.

### 2.1 Search strategy

The components of the search strategy, namely the search phrases, search engines, and search strategy, will be elaborated upon in the following sections.

#### 2.1.1 Search terms

The methodology of Kitchenham et al. [21] served as the basis for selecting the search phrases used in this study.

1. The process of identifying relevant keywords from scholarly papers.
2. Employing alternate synonyms for the identified keywords.
3. Generating key terms based on the research questions.

The search method employed in this study was the utilization of specific keywords and phrases such as "vaccine acceptance," "novel coronavirus," "vaccines," "vaccination," "COVID vaccine," "vaccine hesitancy," "COVID-19," "COVID vaccine acceptance," "Ghana," and "level of COVID-19 vaccine acceptance and vaccine hesitancy." The study followed a specific procedure when querying search engines, using the following terms: (i) COVID AND vaccine AND acceptance; (ii) COVID AND vaccine AND hesitancy; (iii) level AND of AND COVID-19 AND vaccines AND acceptance; and (iv) novel AND COVID-19 AND hesitancy. The researchers (GBA and RAH) designed the search string to balance manageability and sufficient coverage.

#### 2.1.2 Search engines

The researchers (GA, ABD, and DKA) undertook the selection process to identify suitable and relevant search engines after establishing the search terms. The researchers did not limit their search engine selection based on their home institution’s accessibility. We searched several databases, including PubMed, SpringerDirect, ProQuest, MedLine, WorldCat Discovery, Embase, EBSCOhost, and ScienceDirect, to identify primary studies. The study used the generated search strings to specifically target articles in the aforementioned databases. The study further refined the search to include the period from January 1, 2020, to April 2023, inclusive. The researchers implemented this temporal restriction to explore the most recent advancements and patterns related to the acceptance and hesitancy surrounding COVID-19 in Ghana.

#### 2.1.3 Search process

The study conducted a first exploratory search to evaluate the adequacy of available literature resources for the study area. After identifying and defining the search engines and search strings, we conducted a thorough search on each of the eight electronic databases individually to retrieve relevant articles. We downloaded and then exported the literature resources from the search into Excel. The study used the software program JabRef (https://www.jabref.org/) as an alternative to Excel for ScienceDirect. This decision was motivated by the electronic database’s lack of an export-to-Excel feature and its limited capacity to download and export only 100 articles at a time. Eventually, the study combined and exported the downloaded articles to Excel for a human review and selection process to identify the relevant ones. The researchers (GA, ABD, and DKA) employed the software package Mendeley (https://www.mendeley.com/) to save and manage relevant articles. Moreover, we limited the search parameters to only English-language articles and limited the geographical scope to only retrieve materials from Ghana.

### 2.2 Study selection

**Fig 1:**
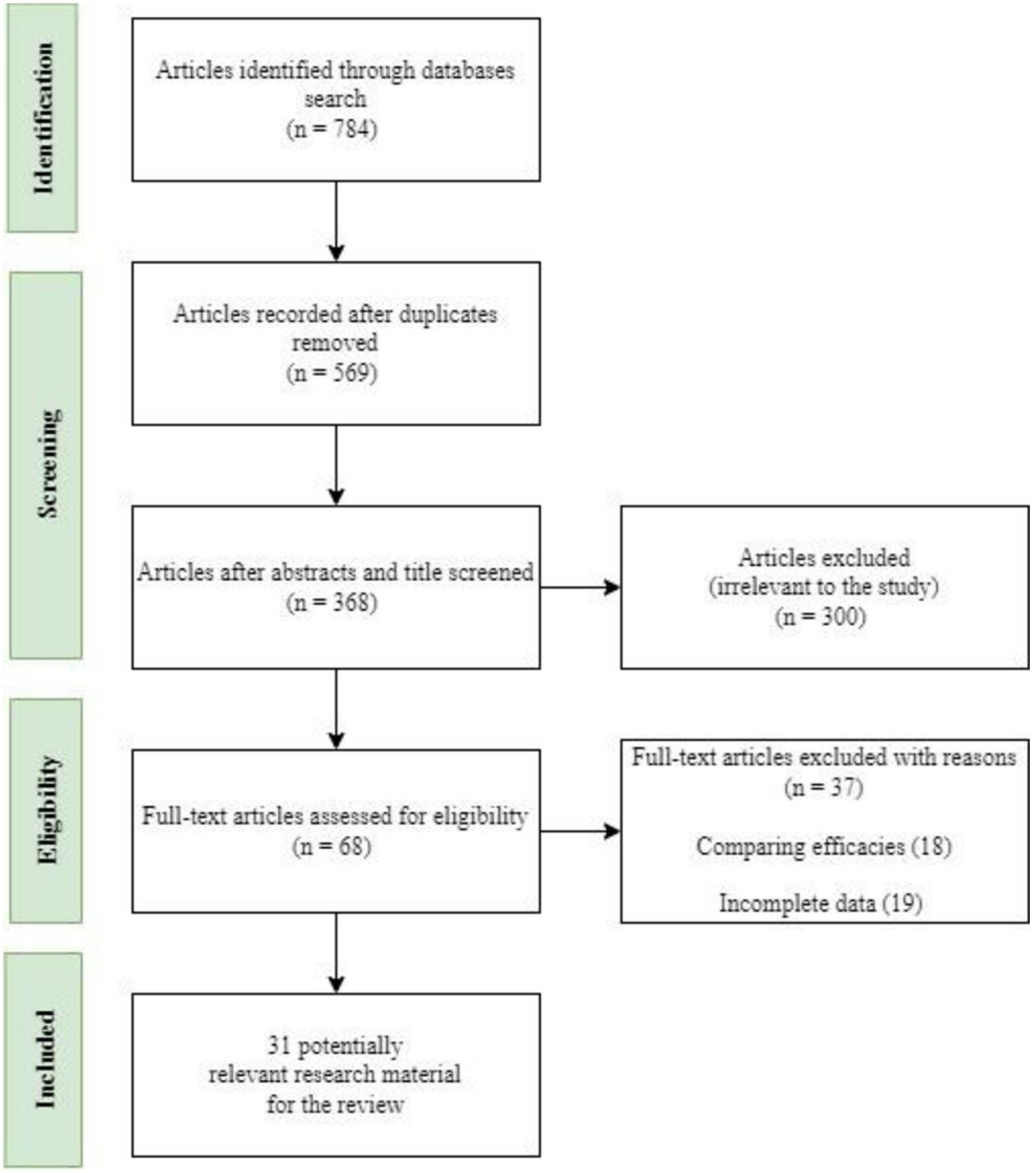
PRISMA flow diagram for eligible article identification.

The study selection process excluded articles that did not offer relevant information for addressing the research questions outlined in this review. Researchers (GBA, RAH, ABD, and DKA) carried this out. The selection process consisted of two parts. We assessed papers in the first phase, known as selection stage 1, to determine their relevance in addressing the study questions. The researchers removed any irrelevant articles from consideration using predetermined inclusion and exclusion criteria. The researchers used selection criteria in the second stage of the process to identify relevant and high-quality papers. The search process yielded a total of 784 articles. The articles underwent a process of selection and elimination, which involved scrutinizing the titles, removing any duplicates, selecting potentially relevant papers based on abstract scrutiny and inclusion criteria, and conducting a thorough review of the selected papers for quality assessment. The review approach selected relevant research papers of satisfactory quality and subsequently utilized them for data extraction. The researchers implemented the search criteria based on the specified research questions to identify appropriate papers for inclusion in the systematic review. Fig 1 depicts the search methodology and the progressive count of publications identified at each stage.

#### 2.2.1 Inclusion criteria

The procedure includes the inclusion of articles sourced from academic journals, including but not limited to the following:

1. Articles with the primary goal of examining the factors that contributes to COVID-19 vaccine acceptance and hesitancy.
2. Articles published between 2021 to April 2023 were included.
3. Articles used in the study should focus on Ghana.
4. Papers written in English were included.

#### 2.2.2 Exclusion criteria

Excluded from consideration were papers that consisted just of an extended abstract or a PowerPoint presentation.

1. Books and magazines were omitted.
2. In addition, review papers were omitted from consideration, resulting in a total of 68 articles that were potentially relevant after undergoing a quality assessment evaluation. Following the evaluation of eligibility based on the 68 articles, a total of 31 final relevant studies were identified.

### 2.3 Study quality assessment

**Fig 2:**
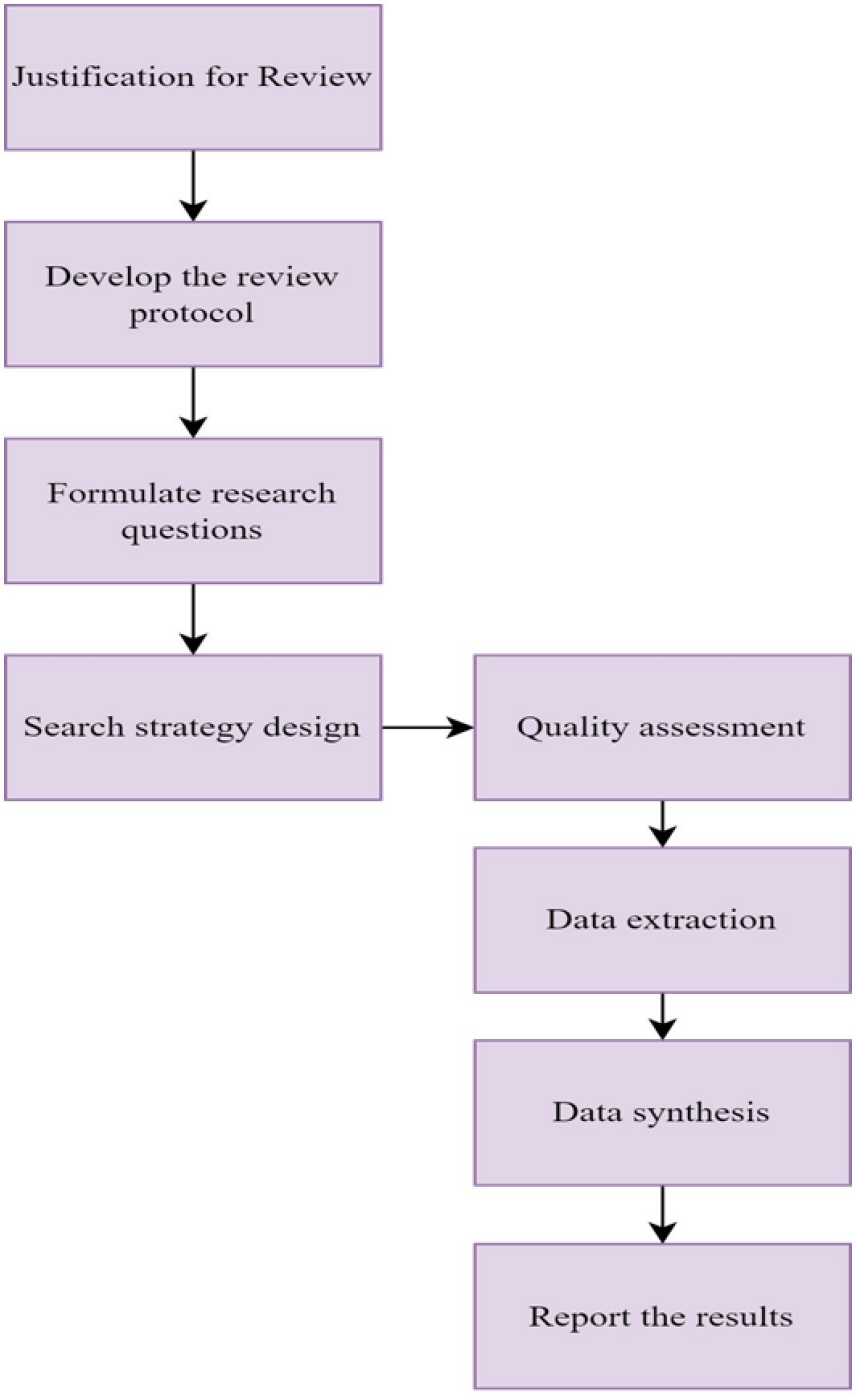
The review protocol.

The researchers GBA and RAH implemented measures during the evaluation process to ensure the quality of the search. After conducting an initial automatic search, we manually reviewed the articles. Subsequently, we conducted a thorough examination and analysis of the titles and abstracts to ascertain the suitability of the papers for inclusion or exclusion. We conducted the initial search for articles in private browsing mode to mitigate the potential impact of prior search history. Moreover, the study developed a comprehensive questionnaire to assess the relevance of the incorporated studies. The purpose of formulating these questions was to assess the pertinence, thoroughness, and reliability of the papers. Wells and Littell [28] guided the formulation of several of these questions. We assigned a score to each question based on one of three alternative answers: "yes" with a score of 1, "partly yes" with a score of 0.5, and "no" with a score of 0. The cumulative scores were obtained by aggregating the scores derived from responses to the quality evaluation questions, representing the quality score assigned to each of the studies included in the analysis. The researchers only included papers with a quality score of 5 or higher in the data extraction and synthesis processes. Fig 2 illustrates the review protocol and Table 3 presents the quality assessment questions.

**Table 3:** Quality assessment questions.

#	Question
Q1	Are the aims of the study reported?
Q2	Are the acceptance rate stated?
Q3	Are the factors that lead to COVID-19 vaccine acceptance stated?
Q4	Are the factors that lead to COVID-19 vaccine hesitancy stated?
Q5	Is the study period for the research specified?
Q6	Is the region or location in which the study was carried out clearly stated?
Q7	Is the population type of the study written?
Q8	Are the study design clearly stated?
Q9	Is the demographic variable that has a correlation with vaccine acceptance or hesitancy stated?
Q10	Are the results and findings clearly stated?
Q11	Are the limitations of the study specified?

### 2.4 Data extraction

The study created a Microsoft Excel data form to systematically collect data that addresses the review questions outlined in each included paper. Researchers GBA and RAH carried out the data extraction. We accomplished this by utilizing Table 2. The study has compiled a summary of data on COVID-19 vaccine acceptance, COVID-19 vaccine hesitancy, acceptance rates, study duration, and geographical location. The collection process also included gathering standard information such as publication details, publication date, title, author name or names, and publication location. The researchers noted during the data extraction process that not all studies included in the analysis could address all the research questions posed in the review. The presence of varying terminologies across different studies presented an additional challenge during the data extraction process. In certain academic works, the terms "novel covid19" and "SARS-CoV-2" have occasionally been employed as substitutes for the term "COVID-19." To mitigate any problems related to ambiguity, we have chosen to utilize the term "COVID-19" for all.

### 2.5 Data synthesis

The researchers, GBA and RAH, saved the collected data for use in the data synthesis step. The primary goal of data synthesis is to consolidate and summarize the data from the included studies to provide comprehensive responses to the established review questions. The study added up the findings from all the included studies that offer comparable or equivalent evidence to arrive at a conclusion. The study extracted quantitative data for the review and subsequently synthesized them to present our findings in a comparable manner [29]. Furthermore, we employed a narrative synthesis approach in response to the data obtained from our review questions. Consequently, the researchers employed a variety of visualization approaches, such as doughnut charts, clustered bar graphs, line graphs, clustered columns, and pie charts. Finally, the researchers summarized and illustrated the findings using tables.

### 2.6 Brief overview of the healthcare system in Ghana

The current study provided insight into the location-specific distribution of articles reporting the acceptance rate of the COVID-19 vaccine. Thus, providing a summary of Ghana and its healthcare system is crucial. Ghana generally consists of 16 administrative regions, each further subdivided into a total of 261 districts, and each of these 16 regions has its own Ghana Health Service office [30]. Ghana has four primary classifications of healthcare delivery systems. This consists of the public, private-for-profit, private-not-for-profit, and traditional systems [31]. The Ghanaian Ministry of Health is responsible for supervising the nation’s health system. Health centers and district hospitals provide basic health care; regional hospitals provide secondary health care; and the top two teaching hospitals provide tertiary services. The two teaching hospitals are situated in the Greater Accra Region and Ashanti Region of Ghana.

## 3. Results

### 3.1 Description of the included studies

This section offers a concise summary of the included studies. The study identified a total of 31 scholarly articles on the topic of COVID-19 vaccine acceptance and vaccine hesitancy, covering the period from 2021 to April 2023, inclusive. Table 4 lists the research questions that each of the included papers addresses.

**Table 4:** Research questions answered in the included studies.

Selected studies	RQ1	RQ2	RQ3	RQ4	RQ5
[10]	*		*	*	*
[11]	*	*	*	*	
[12]	*	*	*	*	*
[13]	*	*	*	*	*
[15]		*		*	
[16]		*	*	*	*
[32]	*	*	*	*	*
[14]	*	*	*	*	*
[17]		*	*	*	*
[18]	*	*	*	*	*
[33]	*			*	*
[20]	*	*	*	*	*
[7]	*	*		*	
[19]	*	*		*	
[34]		*	*	*	
[35]		*		*	*
[36]	*	*		*	
[37]	*		*	*	
[38]	*	*		*	*
[39]		*	*	*	
[40]	*	*		*	*
[41]	*		*	*	*
[42]		*		*	
[43]		*		*	
[44]	*			*	
[45]	*	*	*	*	*
[46]			*	*	
[47]				*	
[48]	*	*	*	*	
[49]	*	*		*	*
[50]	*	*		*	

* represents the research questions answered in the included studies.

### 3.2 Publication year

**Fig 3:**
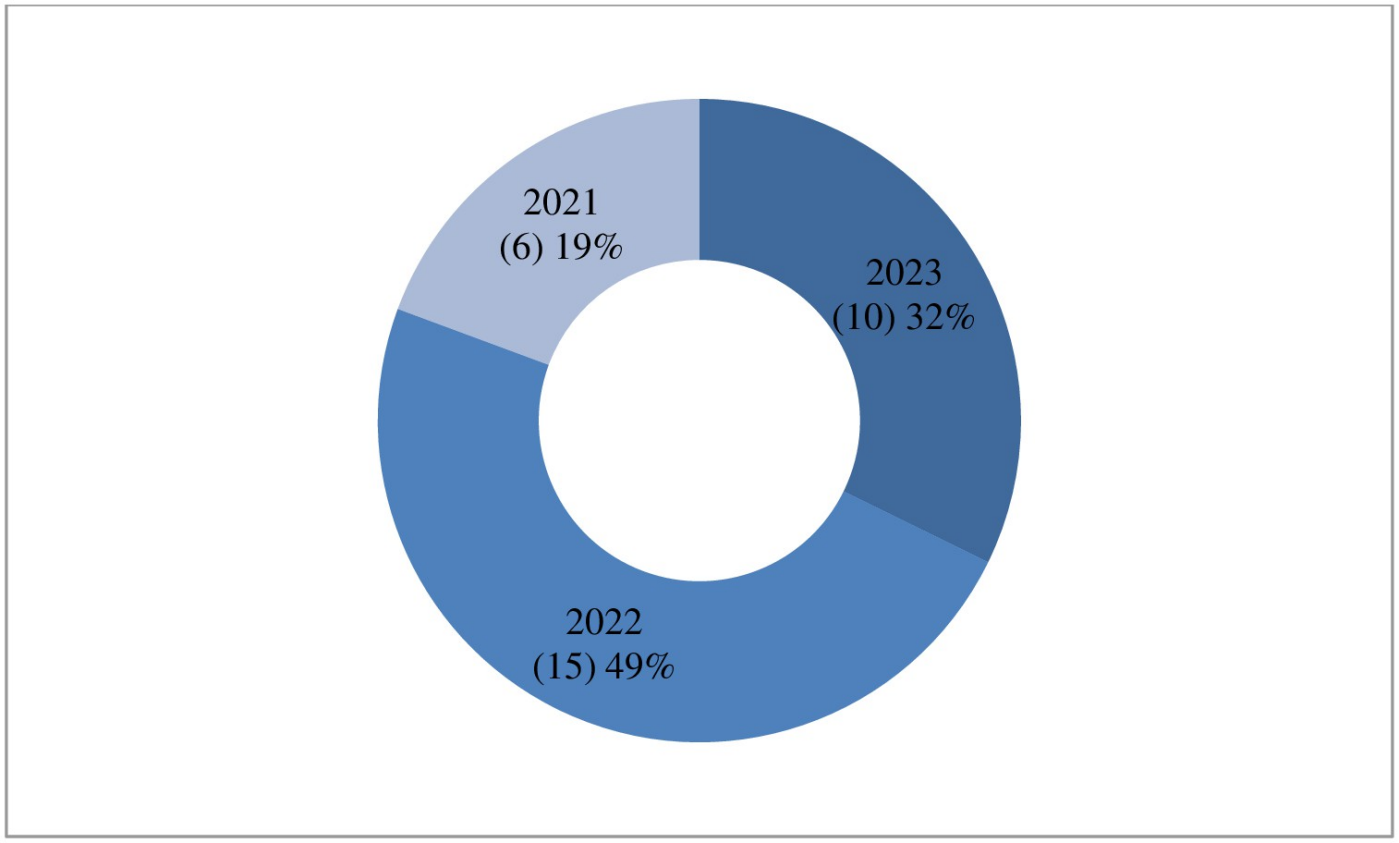
Publication distribution from the year 2021- April 2023.

Fig 3 presents the distribution of articles published from 2021 to April 2023. Overall, the distribution indicates a slight decline in the research trajectory within the field of study. The doughnut charts illustrate a steady rise in the publication of studies that explore the factors influencing the acceptance of COVID-19 vaccines and the reluctance to receive the vaccine. This trend occurred between 2021 and 2022. The findings also observed a slight decrease from 2022 to April 2023.

### 3.3. Publication source

Table 5 provides a summary of the articles, including the number of primary studies, in the respective journal. The studies incorporated in this analysis were published in a total of nineteen distinct academic journals. PLoS One stands out as the journal with the highest number of publications, totaling five. Subsequently, the aforementioned publications included Vaccines (3), Advances in Public Health (3), and Ghana Medical Journal (3).

**Table 5:** Summary of journal publications (2021– April 2023).

Journal name	Frequency
PLoS One	5
Vaccines	3
Advances in Public Health	3
Ghana Medical Journal	3
International Journal of Environmental Research and Public Health	2
Archives of Public Health	2
BMC Infectious Diseases	1
Human resources for health	1
Tropical Medicine and Health	1
Radiography	1
Clinical and Experimental Vaccine Research	1
Human Vaccines & Immunotherapeutics	1
Health Science Reports	1
The Pan African Medical Journal	1
Public Health Challenges	1
Health Research Policy and Systems	1
Therapeutic Advances in Vaccines and Immunotherapy	1
Risk Management and Healthcare Policy	1
African Geographical Review	1

### 3.4 Contributing factors to the COVID-19 vaccine’s acceptance in Ghana

Table 6 outlines the factors that contribute to Ghana’s acceptance of the COVID-19 vaccine, based on the reviewed literature. The findings show that the acceptance of the COVID-19 vaccine in Ghana is primarily attributable to the citizens’ positive perception of the vaccine, the vaccine’s safety, and their confidence in its efficacy. In addition, it emerged that knowledge of COVID-19 and a positive attitude toward the vaccine play a significant role in the acceptance of the COVID-19 vaccine in Ghana. We found several additional factors associated with the acceptance of the COVID-19 vaccine. These factors encompass the aspiration to protect oneself and one’s family, the gender of the participants, the impact of witnessing others receiving the vaccine, the respondents’ readiness for vaccination and their understanding of the virus’s susceptibility, their marital status, their religious affiliation (Christianity), and their financial capacity to cover the vaccine costs. Several other factors significantly influenced Ghana’s acceptance of the COVID-19 vaccination, albeit to a lesser degree. These factors included the perceived advantages of receiving the vaccine. Non-Christian, receiving cost-free vaccines, having attained the highest degree of education, currently receiving the latest vaccines such as Hepatitis B vaccine, employed in faith-based health facilities, having confidence in the government’s implementation of steps to combat COVID-19, employees receiving a salary, individuals who test positive for COVID-19, and having a strong level of trust in the government.

**Table 6:** Reasons for accepting COVID-19 vaccine.

#	Reasons	Studies	Frequency
1	Good perception of vaccine	[10, 38, 40, 41]	4
2	Safety of the vaccine	[11, 18, 19, 49]	4
3	Trust in the effectiveness	[19, 32, 41, 44]	4
4	Knowledge of COVID-19	[14, 36, 50]	3
5	Good attitude towards vaccine	[10–12, 41]	3
6	Protect oneself and family	[14, 40]	2
7	Gender	[20, 36]	2
8	Seeing others get the vaccine	[38, 49]	2
9	Willingness	[46, 49]	2
10	Believing COVID-19 information	[48, 49]	2
11	Perceived susceptibility	[12, 14]	2
12	Relative being diagnosed with COVID-19	[32, 49]	2
13	Being married	[33, 50]	2
14	Receive other forms of vaccination twice	[38, 40]	2
15	Being a Christian	[13, 20]	2
16	Willing to pay for the vaccine	[14, 44]	2
17	Perceived benefit	[12]	1
18	Non-Christian	[13]	1
19	Vaccine being cost-free	[36]	1
20	Highest educational level	[50]	1
21	Receiving recent vaccines such as Hepatitis B vaccine	[10]	1
22	Working in faith-based health facilities	[13]	1
23	Trust in the accuracy of measures taken by government in the fight against COVID-19	[45]	1
24	Salary workers	[33]	1
25	Test positive for COVID-19	[38]	1
26	High trust in Government	[38]	1

### 3.5 Contributing factors to the COVID-19 vaccine’s hesitancy in Ghana

This section provides a summary of the multiple contributing factors to COVID-19 vaccine hesitancy in Ghana throughout the period spanning from 2021 to April 2023. The collected data indicates that the primary factor leading to COVID-19 vaccine hesitancy in Ghana is the presence of adverse side effects associated with the vaccines. Subsequently, a lack of trust in the vaccine, a lack of confidence in its safety, fear, spiritual and religious convictions, and logistical inadequacies emerged as contributing factors to COVID-19 vaccine hesitancy. Also, the results from Table 7 present additional evidence indicating that a lack of sufficient knowledge about COVID-19 among individuals in Ghana, coupled with uncertainties and concerns related to conspiracy theories surrounding the impact of vaccines on the Ghanaian race, doubts regarding the efficacy of the vaccine, concerns about fertility implications, a lack of trust in state institutions, the perception of rapid development and approval of vaccines, prolonged waiting times at vaccination centers, gender disparities, educational attainment, safety concerns, traumatic stress, media attention, and political associations, collectively contributed to vaccine hesitancy in Ghana in relation to COVID-19. Nevertheless, it was found that factors such as residing in less urbanized areas, the politicization of response measures, marital status, disbelief in the existence of COVID-19, perceptions of low susceptibility to the virus, a preference to wait until the vaccine is perceived as safe, a lack of trust in vaccines, and reliance on information from newspapers were also identified as contributing factors to vaccine hesitancy in Ghana. However, we found that these factors had a lesser impact compared to the previously identified factors.

**Table 7:** Reasons for COVID-19 vaccine hesitancy.

#	Reasons	Studies	Frequency
1	Adverse side effects of the vaccines	[14, 18, 20, 32, 34, 36, 38, 40]	8
2	Mistrust in the vaccine	[7, 13, 34, 36, 45]	5
3	Not confident in the safety of the vaccines	[12, 32, 38, 39, 43]	5
4	Fear	[11, 13, 15, 17]	4
5	Spiritual and Religious briefs	[13, 17, 19, 34]	4
6	Insufficient logistics	[7, 35, 38, 49]	4
7	Not having enough information	[34, 40, 45]	3
8	Uncertainty	[13, 17, 43]	3
9	Conspiracy theory & concerns about its effect on the Ghanaian race	[7, 18, 35]	3
10	Doubt about the vaccine’s efficacy	[18, 38]	2
11	Fertility concerns	[12, 18]	2
12	Lack of trust in state institutions	[17, 47]	2
13	Vaccines were rapidly developed and approved	[19, 20]	2
14	Long queues at vaccination centers	[36, 42]	2
15	Gender	[13, 17]	2
16	Educational Level	[13, 17]	2
17	Safety concerns	[13, 17]	2
18	Traumatic stress	[11, 48]	2
19	Media attention	[15, 43]	2
20	Political connotations	[17, 19]	2
21	Residents of less urbanized regions	[17]	1
22	Politicization of response measures	[47]	1
23	Marital Status	[17]	1
24	Belief that COVID-19 doesn’t exist	[12]	1
25	Perceptions of not being susceptible to COVID-19	[14]	1
26	Want to wait for a while until it seems safe to take the vaccine	[12]	1
27	Lack of belief in vaccine	[34]	1
28	Obtaining information from newspaper	[15]	1

### 3.6 Demographic factors

The significance of demographic analysis lies in the provision of valuable information that can inform effective decision-making processes undertaken by governmental bodies and social service organizations [51, 52]. Additionally, it aids individuals in comprehending the attributes of a certain population and its potential future transformations, a crucial aspect in informing decision-making processes. The results of the conducted review show that the primary demographic factors influencing the acceptance and hesitancy of COVID-19 in Ghana are educational attainment, gender, religious affiliation, age, marital status, and the primary source of information about the COVID-19 vaccine (see Table 8). It is critical to recognize that the assessment of factors influencing COVID-19 vaccine acceptance and hesitancy in Ghana did not extensively examine demographic factors such as employment status, rural or urban residency, occupation, level of experience, and political party affiliation among opposition voters.

**Table 8:** Demographics factors.

Demographics	Studies	Frequency
Educational level	[10, 13, 16, 18, 37, 39, 41, 45, 46]	9
Gender	[11, 13, 16, 32, 34, 39, 45, 48]	8
Religion	[13, 18, 20, 34, 37, 39, 41, 46]	8
Age	[14, 16, 20, 34, 37, 41]	6
Marital Status	[17, 34, 45]	3
Primary source of information about COVID-19 vaccine	[16, 18, 39]	3
Employment	[10, 45]	2
Rural settlers	[41, 45]	2
Urban dwellers	[39, 41]	2
Occupation	[12]	1
Experience	[20]	1
Opposition political party voters	[39]	1

### 3.7 Study approaches

The findings from the current study further demonstrate that the main research methodology used to study the factors that influence the acceptance and hesitancy of the COVID-19 vaccine in Ghana has predominantly been the quantitative approach, as shown in Table 9. Subsequently, the mixed-methods technique was used. However, the review findings have highlighted the inadequate use of the qualitative approach in studying the factors that influence Ghana’s acceptance and hesitancy towards the COVID-19 vaccine.

**Table 9:** Study approaches used.

Approach	Studies	Frequency
Quantitative	[10–18, 20, 32–34, 37–41, 43, 45–47, 49, 50]	25
Mixed Method	[7, 35, 36, 42]	4
Qualitative	[19, 44]	2

### 3.8 COVID-19 vaccine acceptance rate in Ghana

The review of the COVID-19 vaccine acceptance rate in Ghana from September to October 2020 shows a highly promising rate among healthcare workers [13]. However, a further study conducted from September to October 2020 on citizens above 18 years old in all 16 regions of Ghana shows a marginal decrease [17]. The decline persisted until December 2020. During the first two months of 2021, there was a notable decrease in the rate of acceptance of COVID-19 vaccines among citizens in the 16 regions of Ghana. However, data collected from January to March 2021 show a high rate of acceptance of the COVID-19 vaccination (Kintampo North-Bono East Region). From February 2021 to June 2021, there was a sustained decline in the acceptance rate. It is imperative to recognize that between May and July 2021, there was a significant increase in the rate of acceptance of COVID-19 vaccines. Furthermore, it is noteworthy that the period spanning June to July 2021 had the highest reported level of acceptance of COVID-19 vaccines. However, there was a decrease in the COVID-19 vaccine acceptance rate in September 2021. In contrast, November had a slight increase in vaccine acceptance rate compared to September 2021. It is important to acknowledge that there was a decrease in the rate of acceptance of COVID-19 vaccines from January to February 2022. However, it is noteworthy that by November 2022, there had been a significant increase in the acceptance rate of COVID-19 vaccines in Ghana (see Table 10).

**Table 10:** Data collection period vs. acceptance rate.

Selected Studies	Data Collection Period	Acceptance Rate (%)	Population Type	Area
[13]	September-October 2020	70	Health care workers	16 Regions in Ghana
[17]	September- October 2020	65	Citizens above 18 years	16 Regions in Ghana
[33]	October—December 2020	54.1	Adult Ghanaians	16 Regions in Ghana
[32]	January- February 2021	39.3	Health care workers	16 Regions in Ghana
[49]	January- March 2021	78.6	Health care workers	Kintampo North
[41]	January- March 2021	79	Adult Ghanaians	16 Regions in Ghana
[18]	February 2021	59.3	Registered Radiographers	16 Regions in Ghana
[16]	February 2021	21	Adult Ghanaians	16 Regions in Ghana
[45]	March- June 2021	17.5	Citizens above 18 years	16 Regions in Ghana
[12]	May- July 2021	62.7	Adult Ghanaians	Dodowa
[14]	June- July 2021	82.6	OPD attendants	Bono Region
[35]	June- September 2021	32.5	Government Officials and community leaders	Volta Region
[10]	May- November 2021	41.9	Residents in Rural Communities	Northern Region/ Ashanti Region/ Western North Region
[40]	September- November 2021	73.3	Parents and Guardians	16 Regions in Ghana
[14]	January- February 2022	46.2	Citizens above 18 years	16 Regions in Ghana
[20]	November 2022	73.6	Health care workers	Bono Region

Table 9 illustrates that the main population group targeted for measuring the acceptance rate of COVID-19 vaccines in Ghana consisted predominantly of individuals aged 18 years and older, as well as healthcare professionals. It is worth noting that the population also included registered radiographers, OPD attendants, government officials, community leaders, residents in remote communities, and parents and guardians.

The results show that the majority of the studies covered in detail all 16 regions in Ghana. Importantly, the study included studies from specific regions in Ghana, such as Kintampo North, Dodowa, Bono Region, Volta Region, Northern Region, Ashanti Region, and Western North Region.

## 4. Discussion

The systematic review yielded conclusive evidence that the citizens of Ghana were inclined to accept or had already accepted the COVID-19 vaccine due to positive perceptions, the vaccine’s safety, trust in its effectiveness, knowledge of COVID-19, and a positive attitude towards the vaccine. This is consistent with the claims made by Forkuo et al. [14] that vaccine acceptance depends on understanding COVID-19 and the vaccines associated with it. Similar to the claims made by Amponsah-Tabi et al. [10], variables such as the highest level of education, salaried employment, high levels of trust in the government, and recent administration of vaccines, including the hepatitis B vaccine, did influence individuals’ acceptance of the COVID-19 vaccine, but their impact was relatively insignificant. The frequency level of these factors was evident in the reviewed studies (see Table 6).

The study shows that several factors contribute to individuals’ hesitancy in accepting the COVID-19 vaccine in Ghana. Individuals in Ghana are hesitant to accept the COVID-19 vaccine due to various reasons, such as unfavorable side effects, doubts about the vaccine’s effectiveness, uncertainty about its safety, fear, religious and spiritual beliefs, logistical difficulties, inadequate information, ambiguity, conspiracy, and worries about the vaccine’s influence on the population. These factors significantly contribute to the hesitancy among individuals in Ghana regarding the acceptance of the COVID-19 vaccine. The contributing factors associated with vaccination hesitancy in relation to COVID-19, as identified in the conducted review, align with the findings of Diaz et al. [53]. The aforementioned study highlights that individuals who had not received the vaccine expressed concerns regarding potential long-term harmful effects that are currently unknown. Given their perspective, these individuals held the belief that COVID-19 vaccines may have adverse effects on reproductive health and fertility and expressed uncertainty regarding their potential influence on fertility. According to Freeman et al. [54], there is evidence suggesting that hesitancy is more prevalent among those who identify as female, have lower income levels, and belong to certain ethnic groups. The findings also support Alhassan et al. [17] conclusion that if not adequately addressed, fear, uncertainty, conspiracy theories, and safety concerns pose significant barriers to the vaccine’s effective adoption.

Information about demographics enables researchers to discern trends and patterns within a population [55]. Within the health sector, it aids in the generation of insights and an understanding of health disparities that arise from factors such as age, gender, disability, or ethnicity [56, 57]. This includes those who face difficulties in accessing healthcare services. The present study identified educational level, gender, religion, age, and marital status as the primary demographic factors commonly assessed when determining the factors that influence the acceptance and hesitancy of the COVID-19 vaccine in Ghana. This was consistent with the findings from Mohammed et al. [20], who found that some factors, including those aged 25 to 45, individuals over 45, males, and Christians, significantly predicted the likelihood of receiving the COVID-19 vaccine. Senior citizens in Ghana, according to Amo-Adjei et al. [19], likewise show less hesitation to get vaccinated, and men are more likely than women to show reluctance to get vaccinated. This assertion is in direct opposition to the conclusions reached by De Figueiredo et al. [58], who reported that socio-demographic characteristics had minimal impact on the increased acceptability of the COVID-19 vaccine.

It is essential to acknowledge that demographic factors such as employment status, rural or urban residence, level of experience, and political party affiliation have been found to have a minimal impact on the acceptance and hesitancy of the COVID-19 vaccine in Ghana. The results from Backhaus [59] are conflicting; they indicated that living in an urban area was positively and significantly associated with the possibility of refusing COVID-19 vaccines, but age and financial resources were associated with a lesser likelihood of doing so. Their study also contended that fear of side effects significantly increased with advancing age, but exposure to negative information regarding vaccines was significantly less prevalent among women with higher levels of education. These results highlight the significance of capturing demographics in research and emphasize the importance of giving sufficient attention to all identified demographics in the current study.

The findings from the review carried out show that most research in this field uses a quantitative approach, with a smaller number opting for a mixed-methods approach. Nevertheless, it is crucial to acknowledge that the number of studies that employed the qualitative approach was inadequate. Even though quantitative research is believed to provide strong evidence that supports evidence-based healthcare practices by guiding clinical decision-making, influencing policy development, and optimizing resource allocation, it will be crucial for future research in this field of study to also employ a qualitative approach [60]. The rationale for conducting qualitative research is its ability to shed light on the intricacies of health behaviors, articulate the lived experiences of illness, formulate effective health interventions, and advance healthcare theories [61]. We cannot overlook the richness of the data provided by the qualitative research approach, as well as its descriptions and depth of inquiry. The mixed method is also a significant approach. According to Wasti et al. [62], effectively combining quantitative and qualitative approaches, as well as their respective datasets and conclusions, needs not only individual talents in each method but also a skill set to integrate them most properly. Therefore, health researchers must carefully consider the optimal method for developing, executing, analyzing, and integrating data that is qualitative and quantitative. They should also focus on presenting their findings in a manner that provides deeper insights and improves their practicality.

The reviewed studies on COVID-19 vaccine acceptance in Ghana show a high acceptance rate of 79% among adult Ghanaians across all 16 regions. Given that Ghana’s adult population was among the first to receive the COVID-19 vaccine, this finding was not surprising. This occurred during the early stages of the vaccine campaign, specifically between January and March 2021. The acceptance rate of 73.3% followed and continued to decrease to 17.5% for the studies, including all 16 regions of Ghana, throughout the data collection period of March—June 2021. The low vaccine acceptance rate during that era was due to the lack of trust among most Ghanaians in the system’s ability to monitor the vaccine’s side effects or adverse effects [45]. The studies that did not include all 16 areas in Ghana reported a COVID-19 acceptance percentage ranging from 82.6% among OPD attendance to 32.5% among government officials and community leaders.

The current study also has some significance, limitations, and implications. This is the first study to examine the factors influencing Ghanaian acceptance and hesitancy toward the COVID-19 vaccine in one extensive review. We conducted the systematic review by searching seven databases for published articles in English and tracking references to relevant studies to prevent any omissions. Furthermore, the study included rigorously verified methods and models that effectively facilitated the attainment of the research objectives. The literature examined in this review consisted of publications that covered the majority of Ghana’s 16 regions and were highly representative.

The present study’s limitation stemmed from its exclusive analysis of English-language publications. Consequently, the exclusion of additional articles in other languages that explore the factors affecting the acceptance and hesitancy of the COVID-19 vaccine in Ghana may limit the capacity to draw thorough conclusions. The review was focused on Ghana. Therefore, the results may not provide a fair representation of the cultural, social, and geopolitical aspects that affect the acceptance and hesitation of the COVID-19 vaccination on a global or regional scale. Furthermore, the majority of publications included in the current analysis were quantitative, and different studies utilized various questionnaires or surveys. This lack of uniformity may have led to response bias.

The study also has practical implications for policymakers, healthcare practitioners, and community leaders in Ghana. The current review findings provide empirical evidence to justify the start of further research on Ghana’s acceptance and hesitancy of COVID-19 vaccines. The study also provides healthcare practitioners with an approach for identifying country-specific characteristics that influence the acceptance and hesitancy of vaccines. In future situations, this approach may be used to devise strategies that facilitate the extensive and equitable distribution of vaccines across Ghana. This study will greatly benefit health policymakers by assisting them in developing evidence-based promotional strategies to enhance public engagement in the COVID-19 vaccine roll-out. Furthermore, now that COVID-19 is no longer considered a pandemic, the study’s findings will assist policymakers in future vaccine rollouts by addressing key factors that influence vaccine acceptance and hesitancy. To combat vaccine hesitancy and encourage vaccination adoption, policymakers may address public disinformation, foster public trust in the vaccine, and promote positive perceptions of its safety and effectiveness. Community leaders should prioritize addressing individuals’ worries about conspiracy theories and the potential impact of vaccines on the Ghanaian race, as shown by the research results. Community leaders should use the chance to readily embrace vaccines in public settings, fostering a culture of acceptance among other community members. If there are concerns among individuals regarding the potential impact of vaccines on fertility, community leaders can enlist the assistance of local health practitioners to provide education and information to the community.

## 5. Conclusion

The study concluded by identifying twenty-six (26) factors that were critical in determining Ghana’s acceptance of the COVID-19 vaccine. Key determinants among these factors included a favorable perception of the vaccine, trust in its safety and effectiveness, sufficient knowledge about COVID-19, a positive attitude towards vaccination, the motivation to protect oneself and one’s family, gender, witnessing others receiving the vaccine, willingness to be vaccinated, and confidence in the accuracy of COVID-19-related information. Therefore, to enhance the level of acceptance of the COVID-19 vaccine in Ghana, it is crucial to meticulously consider these identified pivotal factors while educating people about the need to accept the COVID-19 vaccine. This is important because the Ghana Health Service, in particular, has recently initiated steps to help individuals who have not yet received the vaccine. The primary factors contributing to COVID-19 vaccine hesitancy in Ghana encompassed twenty-eight (28) causes, with the most significant including unfavorable vaccine side effects, distrust towards the vaccine, insufficient confidence in its safety, fear, spiritual and religious beliefs, logistical inadequacies, lack of information, uncertainty, conspiracy theories, concerns about its impact on the Ghanaian population, doubts regarding the vaccine’s effectiveness, and worries about its effects on fertility. Policymakers and stakeholders should consider these identified factors that influence vaccine hesitancy in their efforts to increase public awareness and acceptance of the COVID-19 vaccine. This is crucial for effectively controlling the pandemic and preparing for any future outbreaks in Ghana. Furthermore, now that COVID-19 is no longer considered a pandemic, we must conduct additional studies. Such studies should assist in analyzing the factors that are still causing many individuals to hesitate to get the vaccine, as indicated by the data provided in the study. Additionally, these studies should thoroughly consider the essential demographic parameters commonly evaluated when studying the factors influencing acceptance and hesitation towards COVID-19 vaccines in Ghana. Furthermore, these studies should use a qualitative or mixed-methods approach.

## Supporting information

S1 Appendix Vaccine doses received by member states by types as of 20/05/2024. (DOCX)

S1 Checklist PRISMA 2020 checklist. (DOCX)
