# Supplementary material for: COVID-19 vaccine acceptance and hesitancy in Ghana: A systematic review
Source: PLoS One. 2024 Jun 25;19(6):e0305993. doi: 10.1371/journal.pone.0305993 (PMC11198846; doi:10.1371/journal.pone.0305993)
Supplement: S1 Appendix — (DOCX) [file pone.0305993.s001.docx]

**Appendix**

**Vaccine Doses Received by Member States by Types as of 20/05/2024**

| Member State | AstraZeneca | Sinopharm | Sputnik V | Pfizer BionTech | J&J | Sinovac | Moderna | Sputnik light | Covaxin |
| --- | --- | --- | --- | --- | --- | --- | --- | --- | --- |
| Algeria | 2,865,600 | 700,000 | 1,000,000 |  | 6,508,800 | 22,975,200 |  |  |  |
| Angola | 7,208,440 | 15,089,622 | 588,550 | 10,571,310 | 4,972,050 |  | 4,489,440 |  |  |
| Benin | 278,400 |  | 100,000 | 1,107,990 | 3,665,850 | 1,253,400 | 550,800 | 100,000 |  |
| Botswana | 574,560 |  |  | 1,108,080 | 1,396,800 | 804,494 |  |  | 130,000 |
| Burkina Faso | 246,800 | 1,074,400 |  | 2,704,230 | 5,600,450 | 600,000 | 376,800 |  |  |
| Burundi |  | 500,000 |  |  | 302,400 |  |  |  |  |
| Cameroon | 762,850 | 1,200,000 |  | 264,420 | 6,010,400 |  |  |  |  |
| Cape Verde | 604,200 | 50,000 |  | 433,260 |  |  | 100,100 |  |  |
| Central African Republic | 160,160 | 200,000 |  |  | 3,074,700 | 100,620 | 91,200 |  |  |
| Chad |  | 200,000 |  | 483,210 | 11,465,650 |  |  |  |  |
| Comoros | 18,000 | 400,000 |  | 2,003,190 |  |  |  |  |  |
| Congo Republic |  | 760,000 | 12,000 | 809,500 | 1,772,800 |  |  | 102,006 |  |
| Cote d`Ivoire | 4,671,340 | 8,994,400 |  | 12,879,090 | 7,092,400 |  |  |  |  |
| Djibouti | 175,200 | 1,100,000 | 100,000 | 74,880 | 468,450 | 300,000 |  | 100,000 |  |
| DR Congo | 1,817,000 | 400,000 |  | 5,828,310 | 26,382,200 | 2,400,000 | 3,572,340 |  |  |
| Egypt | 29,190,320 | 6,950,000 | 393,000 | 29,770,090 | 15,513,450 | 20,600,000 | 7,254,980 |  |  |
| Equatorial Guinea |  | 820,000 |  |  | 400,000 |  | 60,000 |  |  |
| Eswatini | 360,800 |  |  | 250,380 | 302,400 |  |  |  |  |
| Ethiopia | 6,981,190 | 19,390,400 |  | 12,061,260 | 42,062,150 | 600,000 | 300,000 |  |  |
| Gabon | 350,000 | 500,800 | 20,000 | 420,030 | 1,413,600 |  | 640,620 |  |  |
| Gambia | 89,400 | 210,000 |  | 819,810 | 904,320 |  |  |  |  |
| Ghana | **12,662,020** |  | **21,000** | **9,831,510** | **9,840,000** |  | **1,229,620** |  |  |
| Guinea | 1,001,320 | 2,100,000 | 407,990 | 3,001,650 | 3,388,800 | 1,506,400 | 188,400 |  |  |
| Guinea-Bissau | 669,600 | 300,000 |  |  | 1,094,400 |  |  |  |  |
| Kenya | 13,675,940 | 300,000 | 75,000 | 10,801,260 | 7,414,950 |  | 3,305,720 |  |  |
| Lesotho | 456,000 | 203,000 |  | 696,150 | 2,070,580 |  |  |  |  |
| Liberia | 342,000 |  |  | 1,417,620 | 3,591,444 |  |  |  |  |
| Libya | 1,730,400 | 2,000,000 | 1,461,250 | 1,665,270 | 100,000 | 150,000 |  |  |  |
| Madagascar | 794,990 |  |  | 7,652,970 | 7,209,950 |  |  |  |  |
| Malawi | 2,567,720 |  |  | 2,093,760 | 6,269,170 |  |  |  |  |
| Mali | 597,600 | 600,000 |  | 1,171,260 | 3,333,550 | 2,735,200 | 79,200 |  |  |
| Mauritania | 2,645,350 | 1,738,000 |  | 771,080 | 2,484,000 |  |  |  |  |

**Appendix**

**Vaccine Doses Received by Member States by Types as of 20/05/2024 (cont.)**

| Member State | AstraZeneca | Sinopharm | Sputnik V | Pfizer BionTech | J&J | Sinovac | Moderna | Sputnik light | Covaxin |
| --- | --- | --- | --- | --- | --- | --- | --- | --- | --- |
| Mauritius | 500,800 | 1,605,000 | 250,000 | 387,270 | 439,200 | 4,000 | 200,000 |  | 200,000 |
| Morocco | 8,723,200 | 46,814,000 |  | 4,956,980 | 302,400 |  |  |  |  |
| Mozambique | 20,446,220 | 9,723,278 |  | 9,788,220 | 8,989,700 |  |  |  |  |
| Namibia | 526,600 | 350,000 |  | 432,900 | 676,800 | 100,000 |  |  |  |
| Niger | 1,196,200 | 1,328,800 |  | 640,260 | 7,939,200 | 200,000 |  |  |  |
| Nigeria | 20,741,840 | 470,000 |  | 26,394,660 | 77,927,850 |  | 16,230,960 |  |  |
| Rwanda | 7,428,830 | 1,345,600 | 227,900 | 14,456,600 | 1,234,860 |  | 5,795,100 |  |  |
| Sahrawi Republic | 20,000 |  |  |  |  | 10,600 |  |  |  |
| Sao Tome and Principe | 153,200 | 100,000 |  | 5,850 | 100,800 |  | 112,320 |  |  |
| Senegal | 1,979,260 | 1,609,318 | 10,000 | 1,364,300 | 2,569,500 |  |  |  |  |
| Seychelles | 139,000 | 131,120 | 1,000 | 124,260 |  |  |  |  |  |
| Sierra Leone | 1,308,000 | 440,000 | 10,000 | 3,226,590 | 4,491,360 | 200,000 |  |  |  |
| Somalia | 1,870,560 | 931,600 |  | 2,492,630 | 9,233,300 | 290,400 |  |  |  |
| South Africa |  |  |  | 29,804,030 | 11,373,996 |  |  |  |  |
| South Sudan | 298,520 |  |  | 1,527,900 | 5,982,170 |  |  |  |  |
| Sudan | 4,520,960 | 1,567,600 |  | 6,417,720 | 20,624,300 |  |  |  |  |
| Tanzania | 1,065,600 | 6,578,400 |  | 4,564,800 | 35,226,150 | 1,000,000 | 376,320 |  |  |
| Togo | 919,160 | 211,200 |  | 1,450,800 | 2,620,800 | 1,638,200 |  |  |  |
| Tunisia | 4,286,000 | 150,000 | 50,000 | 4,746,510 | 2,540,800 | 3,650,000 | 2,426,328 | 100,000 |  |
| Uganda | 6,624,480 | 2,060,400 |  | 16,773,340 | 22,547,800 | 6,000,000 | 4,855,320 |  |  |
| Zambia | 1,549,300 | 1,995,200 | 50,000 | 5,436,630 | 12,367,050 |  | 188,400 |  |  |
